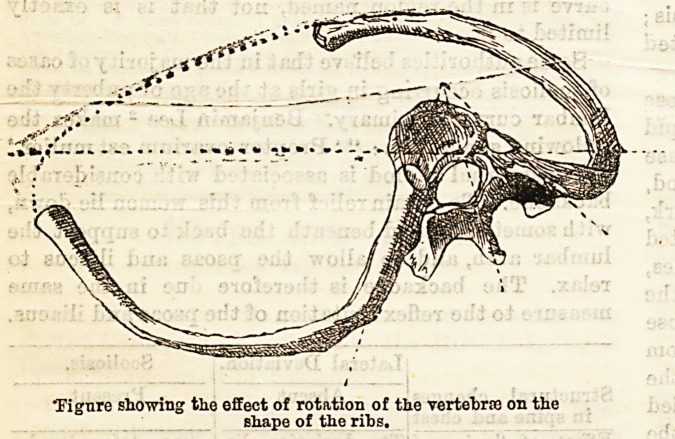# Clinical Aspects of Scoliosis

**Published:** 1895-06-01

**Authors:** A. H. Tubby

**Affiliations:** Assistant Surgeon to the Westminster Hospital; Surgeon to the National Orthopædic Hospital; Surgeon to Out-patients Evelina Hospital for Sick Children


### Medical Progress and Hospital Clinics.

[*The Editor will be glad to receive offers of co-operation and contributions from members of the profession. All letters should be addressed to* The Editor, The Lodge, Porchester Square, London, W.]

### CLINICAL ASPECTS OF SCOLIOSIS.

By A. H. Tubby, M.S.Lond., F.R.C.S.Eng., Assistant Surgeon to the Westminster Hospital; Surgeon to the National Orthopædic Hospital; Surgeon to Out-patients Evelina Hospital for Sick Children.

Before describing the various aspects of true scoliosis, it will be convenient to dispose of lateral deviation, since the two conditions may be, and often are, confused, lateral deviation being called scoliosis; or, a greater error, scoliosis being styled and treated as mere deviation.

**Pure Lateral Deviation**, then, occurs in those whose spinal muscles are weakened, either from too rapid growth at the age of puberty, combined in the case of young girls of the lower class with bad food, want of fresh air, and often excessive physical work, or after acute illnesses. In such cases the segmented spine being insufficiently supported by the muscles, gives way, and insensibly a deviation to one side or the other ensues. Still more likely is it to occur in those patients, who from one cause or another, suffer from inequality of the lower limbs. In many instances, the deviation disappears at once if a cork sole be applied to the boot of the shorter limb. As an instance of the combined effect of heavy trunk and bust with impaired health and overwork, I append notes of the following case, which, commencing as a lateral deviation, subsequently developed into scoliosis, associated with a prominent posterior curvature.

Case.—A. A., aged twenty-two, has been nursemaid for the last five years, and has performed as well the duties of a general servant. She has been accustomed to carry children on the right arm, and for the last three years has noticed that the back has been growing out. There has been considerable pain in the dorsal region, which was temporarily relieved by rest. On examination—she is a very stout girl, the head and trunk being particularly massive. There is no equality in the length of the legs. A long C-shaped curve is seen on the left side extending from the first dorsal to the third lumbar vertebra, with but slight compensatory curve. On suspension these disappeared. She was unable to give up work; and, in spite of supports, a large posterior curve occupying the dorsal region formed, and three years afterwards her condition became steadily worse, an intractable scoliosis resulting.

Fisher[1] has enumerated with much clearness the distinctions between deviation, or lateral "bending" from muscular weakness, and scoliosis, and I place the various points in the accompanying table for comparison.

It is very essential that no error should arise from the lateral deviation of Pott's disease. For the characteristic features of such deviation the remarks in a previous paper in The Hospital may be referred to.

**Scoliosis.**—Having thus excluded simple deviation, let us turn to the varying clinical aspects of scoliosis. Any classification, however convenient, is arbitrary, and cannot cover all the varieties, but many cases may be grouped under the following headings :—

**1 Cases in which the Curvature is Mainly Unilateral, or C Curves.**—The curve may be in the lumbar or dorsal region, or in both. It may be of small extent, or involve the whole dorsal and lumbar regions.

In the former case it is frequently seen in the lumbar region, and results in but little apparent deformity. It is convenient to speak of curve as dorsal and lumbar. These terms merely imply that the chief part of the curve is in the region named, not that is is exactly limited to it.

Some authorities believe that in the majority of cases of scoliosis occurring in girls at the age of puberty the lumbar curve is primary. Benjamin Lee [2] makes the following suggestion: "'Propter ovarium est mulier.' The menstrual period is associated with considerable backache. To obtain relief from this women lie down, with something hard beneath the back to support the lumbar arch, and so allow the psoas and iliacus to relax. The backache is therefore due in the same measure to the reflex irritation of the psoas and iliacus.

| | Lateral Deviation. | Scoliosis. |
|---|---|---|
| Structural changes in spine and chest | Absent | Present . |
| Effect of flexion of the spine ... ... | The deviation disappears | The deformity is more apparent |
| Horizontal position | Ditto | The deformity remains |
| Voluntary muscular effort ... ... | The spine may be straightened temporarily | The spine cannot be completely straightened |
| Raised shoulder and depression just above crest of ilium | On opposite sides of the body | Generally on the same side of the body |
| Suspension ... ... | The deviation disappears | Disappearance of the deformity in initial cases only |

If those muscles on both sides act equally then no effect is produced, but if on one side the muscles are stronger, then curvature ensues." This seems to me far-fetched; but as Bradford and Lovett pertinently say, "Some writers regard the lumbar scoliosis as the chief curve, and as the most common. The question may be regarded as not settled, though for clinical purposes it may be accepted as a fact that the dorsal curve is the one most frequently requiring treatment."

A single curve,[3] or C-shaped curve of large extent, is always of serious import, because of the number of vertebræ implicated. The weight of the head and upper extremities continually tends to increase it, and with the general weakness of the spinal muscles renders treatment lengthy and troublesome. In these cases too, the rotation of the spinous processes is well marked, the secondary effects of the deformity are great, and they are likely to run a more rapid and less favourable course. Such an instance was A. A., quoted above. Generally the curve is in the middle, lower dorsal and lumbar regions; less frequently the upper and middle portions of the dorsal region are affected, *i.e.*, the curve may affect the lower or upper part of the spinal column. A deviation pure and simple may develop into a long C-shaped curve, or more often a double curve forms. Such an instance is the case of F. C., aged 15, who came to me with deviation to the left side, in May, 1892. In November of that year a double curvature had formed, the upper with its convexity to the left, and reaching from the 7th cervical to the 11th dorsal vertebra, and the lower convex to the right, and in the lumbar region.

When the chief curve is very long the resultant distortion of the body is very considerable, inasmuch as the "vertebral column, like the keel of a ship, is the foundation of the structure of the trunk." (Fisher.)

Figure showing the effect of rotation of the vertebræ on the shape of the ribs.

The mechanism may be grasped by attention to the accompanying diagram, in which the effects of the twisting of the vertebra on the ribs are figured. For simplicity let us suppose that the chief convexity is to the right, of large extent, and occupying the dorsal region with a secondary smaller curve to the left in the lumbar region.

The results on the trunk are as follows: (*a*) General appearance. The symmetry of the body is quite lost, the right shoulder being elevated, the left depressed. The right arm is closely approximated to the side, and the left falls away at a considerable angle. The ribs on the right side behind are bulging, while on the left they are depressed. On the right side there is considerable hollowing out of the flank, with prominence of the hip; while on the left the flank is flattened and the hip is less in evidence than usual. This is due to the rotation of the bodies in the lumbar curve to the left, and the consequent pushing backwards of the transverse process on that side, with sinking in of the right process and depression of the muscles covering them. So that, according to the patient's description, the right hip is growing out, whereas it is really the flank which is sinking. If, however, the curve is situated so that it extends over the dorsal and upper lumbar vertebræ, then the prominent hip and hollow flank are on the left, inasmuch as the right transverse processes are rotated backwards in the lumbar region, while those on the left are rotated forwards and the muscles covering them sink in, but be it observed, are not, as a rule, wasted. (*b*) The ribs behind on the right side are prominent with their angles much increased, while in front they are correspondingly depressed, the whole ribs being, as it were, drawn backwards in the planes in which they lie and compressed from before backwards and from right to left. On the concave side the ribs are more prominent anteriorly, but less so posteriorly and the angle is widened. The altered position of the transverse processes sufficiently explains this. On the side of convexity the latter are more prominent than normal and on the concavity the reverse. In addition the right ribs are less oblique, with the intercostal spaces widened, and, if the primary curve affect the lumbar region as well, the distance between the last rib and the crest of the ilium is increased. (*c*) The scapula will be found altered from their natural positions. Thus the right scapula, the out-growing shoulder, is raised, less vertical than usual, and in severe cases almost horizontal, so as to give rise to the idea that its inferior angle is dislocated. It is also farther away from the middle line than normal. The left scapula appears to have sunk, and its position is just the reverse of the right. (*d*) The clavicle on the right side is much curved, and it has been said that in very severe cases of scoliosis even dislocation of the sternal end has occurred. (*e*) The apices of the spinous processes in the dorsal region are twisted to the left, *i.e.*, away from the convexity. But the deviation of these processes is no measure of the deviation of the bodies, which is often much greater, owing to the vertical axis of rotation being situated considerably nearer to the tip of the spinous processes than the fronts of the vertebral bodies. This want of correspondence is an important point to bear in mind when forming a prognosis. (*f*) The transverse processes on the convex side in the dorsal region are prominent and depressed on the concave side. (*g*) As pointed out by Mr. Adams, the height of the spinal column as a whole is decreased owing to the deviation of the bodies to one side or the other, and to the posterior projection of the spinal column, the result of the general yielding. (*h*) The left breast is the more prominent, and the umbilicus is displaced to the left with corresponding fullness on the right side of the abdomen.

Such then are the clinical aspects of a case in which the long curve is mainly dorsal and to the right.

Single curvatures in the cervical region are rare, and seen as the result chiefly of torticollis. But the following case associated with unequal refractive indices of the corneæ is interesting:—

Case.—A. B., aged fifteen, a feeble Jewish boy, came to me at the National Orthopædic Hospital on December 21st, 1893. His general muscular development was bad, the shoulders sloping, the head drooping somewhat, and he was markedly anæmic. He also complained of inability to see clearly. The seventh cervical spine was prominent, and the whole cervical spine deviated to the right. On testing his eyes I found them unequally hypermetropic. The error in the right eye was +3 D, and in the left +5 D. He was fitted with suitable spectacles, and with a change of air, exercise, and under the administration of iron, he improved in general health, and the deviation in the neck disappeared.

(*To be continued.*)

[1] Internat. Encylc. Surg., Vol. VI., pp. 1,067 and 1,069. [2] Trans. Amer. Orth. Assoc., Vol. II., p. 80. [3] For the maintenance of the head in the erect position there must necessarily be small and somewhat abrupt compensatory curves at the extremities of the chief curves.

## Figures and Tables

**Figure f1:**